# Natural Products That Target the Arginase in *Leishmania* Parasites Hold Therapeutic Promise

**DOI:** 10.3390/microorganisms9020267

**Published:** 2021-01-28

**Authors:** Nicola S. Carter, Brendan D. Stamper, Fawzy Elbarbry, Vince Nguyen, Samuel Lopez, Yumena Kawasaki, Reyhaneh Poormohamadian, Sigrid C. Roberts

**Affiliations:** School of Pharmacy, Pacific University, Hillsboro, OR 97123, USA; cartern@pacificu.edu (N.S.C.); stamperb@pacificu.edu (B.D.S.); fawzy.elbarbry@pacificu.edu (F.E.); nguy6477@pacificu.edu (V.N.); lope3056@pacificu.edu (S.L.); kawa4755@pacificu.edu (Y.K.); poor1405@pacificu.edu (R.P.)

**Keywords:** natural products, *Leishmania*, arginase, polyamines, putrescine, spermidine, quercetin, cinnamic acid, green tea polyphenols

## Abstract

Parasites of the genus *Leishmania* cause a variety of devastating and often fatal diseases in humans worldwide. Because a vaccine is not available and the currently small number of existing drugs are less than ideal due to lack of specificity and emerging drug resistance, the need for new therapeutic strategies is urgent. Natural products and their derivatives are being used and explored as therapeutics and interest in developing such products as antileishmanials is high. The enzyme arginase, the first enzyme of the polyamine biosynthetic pathway in *Leishmania*, has emerged as a potential therapeutic target. The flavonols quercetin and fisetin, green tea flavanols such as catechin (C), epicatechin (EC), epicatechin gallate (ECG), and epigallocatechin-3-gallate (EGCG), and cinnamic acid derivates such as caffeic acid inhibit the leishmanial enzyme and modulate the host’s immune response toward parasite defense while showing little toxicity to the host. Quercetin, EGCG, gallic acid, caffeic acid, and rosmarinic acid have proven to be effective against *Leishmania* in rodent infectivity studies. Here, we review research on these natural products with a focus on their promise for the development of treatment strategies as well as unique structural and pharmacokinetic/pharmacodynamic features of the most promising agents.

## 1. Introduction to *Leishmania*

*Leishmania* are protozoan parasites responsible for a spectrum of neglected tropical diseases collectively referred to as leishmaniasis. *Leishmania* are found throughout the tropical and subtropical climates of the world, though the disease is most prevalent in Brazil, China, Ethiopia, India, Iraq, Kenya, Nepal, Somalia, South Sudan and Sudan. Leishmaniasis disproportionately affects populations in rural and impoverished areas and is often linked to environmental damage such as deforestation and urbanization [1,2,3,4,5,6]. Additionally, climate change, civil conflict, war, and human migration have all led to a rise of leishmaniasis cases worldwide [3,5,6]. Each year it is estimated that 1.5 to 2 million new cases occur and due to the lack of an effective vaccine 350 million people residing in 88 countries are at risk for infection [6]. 

At least 20 *Leishmania* species have the ability to infect humans and to cause the various types of leishmaniasis including cutaneous, mucocutaneous, and visceral leishmaniasis [7] and the most common species are listed in Table 1. Visceral leishmaniasis is the most severe form and is lethal when left untreated. The symptoms of visceral leishmaniasis include fever, anemia, and hepatosplenomegaly. The cutaneous and most common form of leishmaniasis involves skin lesions and ulcers. While rare, mucosal leishmaniasis can lead to destruction of mucous membranes of the nose, mouth, and throat.

The parasite is transmitted by female sand flies of the genera *Phlebotomus* and *Lutzomyia*. There are two life cycle forms of *Leishmania*: promastigotes and amastigotes. *Leishmania* promastigotes reside in the gut of the sand fly and are able to infect mammals. When a sand fly bites a mammalian host, the parasites are injected into the skin. After phagocytosis by macrophages, the parasites reside in the phagolysosome, where they transform into the amastigote form. Amastigotes are able to adapt to the acidic and hostile environment of the phagolysosome, to replicate, and to continue to infect new macrophages. *Leishmania* parasites are able to evade destruction and thrive as intracellular parasites by modulating host metabolism and defense mechanisms.

Current therapies used to treat leishmaniasis include agents such as antimonials (sodium stibogluconate (Pentostam) and meglumine antimoniate (Glucantime)), amphotericin B, and miltefosine, but these drugs can be costly, come with significant side effects, and are increasingly becoming side-lined by drug resistance. The need for alternative therapeutics or the coadministration of alternative therapeutics is urgent. Natural products have served as a well-established source for bioactive compounds in drug discovery, thus resulting in a strong interest in the development of natural products to combat leishmaniasis [8,9,10]. The disease is endemic to poor countries and there is little financial incentive for pharmaceutical companies to invest in the production of new drugs. Without proper healthcare infrastructure, natural products as therapeutics are increasingly valuable to people in these areas. Indeed, an estimated 80% of the population in developing countries already depend on traditionally used medicinal plants for their primary health care [11].

## 2. Natural Products as an Antileishmanial Drugs

**Figure 1:**
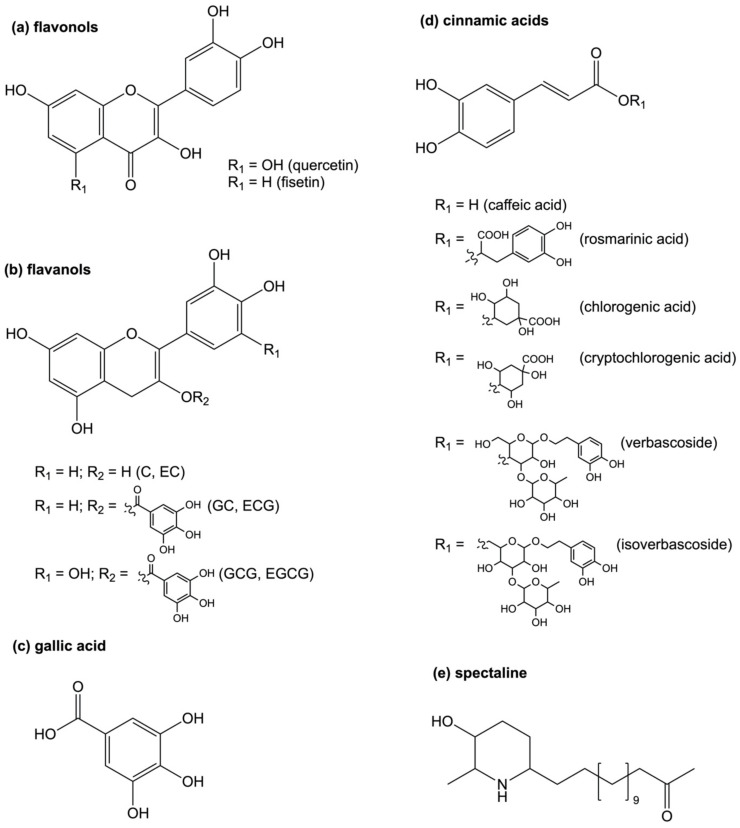
Natural products targeting arginase. Compounds derived from multiple structural classes have been developed as anti-leishmanial agents in drug development including (**a**) flavonols, (**b**) flavanols that give rise to various derivatives based on stereochemistry (**c**), gallic acid, (**d**) cinnamic acid-based analogs, and (**e)** piperidine alkaloids. Abbreviations: catechin (C); epicatechin (EC); catechin gallate (GC); epicatechin gallate (ECG); gallocatechin gallate (GCG); epigallocatechin-3-gallate (EGCG).

Historically, plant components have served as a major source for pharmacologically active agents. Natural products such as plant secondary metabolites play key roles in plant adaptation to the environment, and are capable of acting as herbivore deterrents by eliciting biologic repercussions in the animals that ingest them [12]. It is through this biologic modulation that the potential therapeutic power of these compounds exists, and why plant-based natural products are of notable pharmaceutical interest. According to the U.S. Food and Drug Administration, 34% of new medications approved between 1981 and 2014 were based on natural products or their direct derivatives [13] and similarly about 35% of global medicines are derived from natural products [14]. Therefore, it comes as no surprise that natural products are being considered for the treatment of a wide array of conditions, including leishmaniasis. The high demand for safe, accessible, and effective antileishmanial agents coupled with the fact that natural products serve as a prolific reservoir for drug discovery, make this an exciting and dynamic research field right now. Many of the compounds tested so far target the *Leishmania* arginase, an enzyme of the polyamine pathway. One of the most well-studied classes of natural products in this effort are the phenolic plant secondary metabolites known as flavonoids (Figure 1a,b). This includes flavonols such as quercetin and fisetin, as well as flavanols like those found in green tea, which include catechin (C), epicatechin (EC), epicatechin gallate (ECG), epigallocatechin (EGC), and epigallocatechin-3-gallate (EGCG). While the focus of this review will be on the ability of these flavonoids and other natural products to target the enzyme arginase, it is worth noting that flavonoids likely have multiple targets in addition to arginine metabolism such as tyrosine aminotransferase [15], glycoprotein 63 [16], and trypanothione [17]. 

Flavonoids belong to a wider class of compounds known as polyphenols, which, as their name suggests, contain multiple hydroxyl groups bound to a phenyl ring. Polyphenols are ubiquitous phytochemicals found in many foods and herbal sources representing a highly diverse class of chemical compounds. Like its flavonoid subclass, the larger polyphenol structural class has been investigated for the prevention of numerous indications including cardiovascular disease, neurodegenerative disease, cancer, diabetes mellitus, and osteoporosis [18]. The polyphenolic compounds also hold great promise as antileishmanial agents. Some of the most well-studied polyphenols include gallic acid (Figure 1c) and cinnamic acid derivatives such as rosmarinic, chlorogenic, and caffeic acids (Figure 1d). For example, certain cinnamic acids have been shown to exert their antileishmanial activity through multiple mechanisms that include not only arginase [19], but also less-defined modes such as changes to host and parasite iron homeostasis and parasite mitochondrial integrity [20]. Polyphenols have certainly been shown to be capable of mitigating the damaging effects of oxidative stress by improving the status of related biomarkers in humans [21]. However, the relationship between polyphenols and oxidative stress is highly complex and an active area of investigation [22]. Furthermore, the relevance of oxidative stress biomarkers in the pathogenesis of leishmaniasis remains to be fully elucidated. In other words, it might be overly simplistic to assume polyphenols are working strictly through redox-related mechanisms. For example, it is well-established that soy isoflavones, which are polyphenolic compounds, profoundly affect endocrine function through interactions with estrogen receptors. Altogether, this lends credence to the idea that polyphenolic cinnamic acids derivatives with inherent antioxidant character, are capable of exerting their antileishmanial activity through non-redox mechanisms, such as arginase inhibition [19].

While much work has been done to evaluate the activity of polyphenols against arginase (flavonoids, cinnamic acids, etc.), other structural classes of natural products will also be discussed in this review. These include plant extracts from *Senna spectabilis*, *Sambucus ebulus*, and *Urtica dioica*. These are often complex mixtures that include some combination of flavonoids, phenols, lectins, and alkaloids. For example, spectaline is an alkaloid isolated from *Senna spectabilis* that has been investigated for its anti-leishmanial activity and offers a unique alternative structure to the numerous polyphenols presented in this paper (Figure 1e). This review article will focus on the aforementioned natural products that target arginase in *Leishmania* and discuss their efficacy in vitro and in vivo with a focus on their therapeutic promise as future antileishmanial agents.

## 3. Arginase as a Promising Therapeutic Target

The enzyme arginase has recently received much attention for its role in numerous disease states, such as pathologies of the cardiovascular system, kidney, and central nervous system, dysfunction of the immune system, cancer, and infectious diseases [23,24,25]. In humans, arginase competes with nitric oxide synthase for the amino acid arginine. An increased arginase activity with the associated reduction in nitric oxide appears to be a key contributor for the above listed disease states and numerous studies have investigated the beneficial effect of arginase inhibitors in vitro and in clinical studies (summarized in [23,24,25]). In *Leishmania* parasites, arginase has similarly been a recent focus of research (reviewed in [26,27,28,29,30,31,32]).

**Figure 2:**
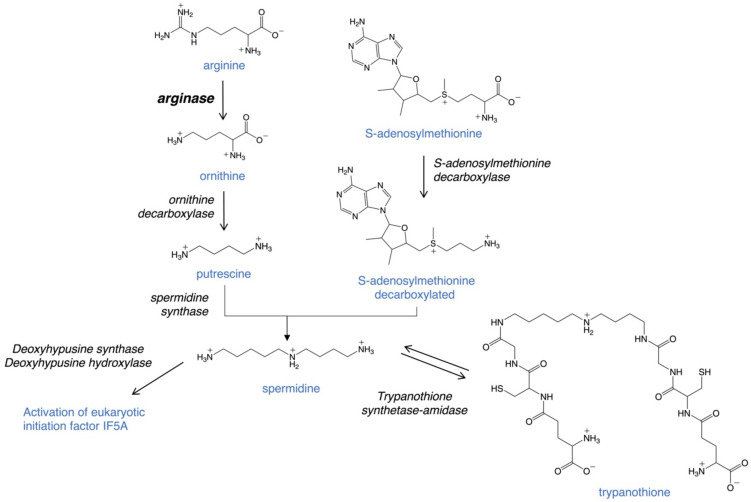
The role of arginase in the polyamine pathway of *Leishmania* parasites. Arginase is the first step in polyamines biosynthesis.

Arginase is the first enzyme of the polyamine pathway in *Leishmania*, which includes the precursors arginine and ornithine and the polyamines putrescine and spermidine (Figure 2). Critical downstream reactions are the hypusination and activation of eukaryotic initiation factor 5A (eIF5A) and the formation of trypanothione, which is essential for defense against oxidative stress. Recent studies have highlighted the importance of polyamines and trypanothione for parasite proliferation and infectivity and validate the pathway as a potential therapeutic target [26,29,30,31,32,33,34,35,36,37,38,39,40]. In particular, arginase as the first enzyme of this pathway has been of considerable interest [26,29,31,32,35,36,39].

Polyamines are ubiquitous polycations that play critical roles in a variety of key processes, including growth, differentiation and macromolecular synthesis. In *Leishmania* parasites, arginase catalyzes the conversion of the essential amino acid arginine to ornithine (Figure 2). Unlike in mammalian host cells, which contain two arginase enzymes, only one arginase gene exists in *Leishmania* and the sole essential role of ornithine is as precursor for polyamine biosynthesis [33,35,39,41]. Ornithine is converted to the polyamine putrescine by the enzyme ornithine decarboxylase and putrescine is further metabolized to spermidine by spermidine synthase. Decarboxylated S-adenosylmethionine, generated by S-adenosylmethionine decarboxylase, donates the aminopropyl group for spermidine formation. Both putrescine and spermidine are positively charged under physiological conditions. Polyamines are essential for parasite viability and as precursors for the formation of trypanothione and hypusine. Trypanothione is a unique thiol formed in trypanosomatids by conjugating two molecules of glutathione to spermidine. The bifunctional enzyme trypanothione synthetase-amidase catalyzes the biosynthesis and hydrolysis of trypanothione [42]. This unique thiol is vital for maintaining the intracellular redox balance and for defense against oxidative stress [28,43,44,45]. In addition, spermidine is the precursor for the hypusination and activation of eIF5A, which is essential for both *Leishmania* parasites and in the mammalian host [46,47,48,49].

Recent studies have highlighted the importance of the polyamine biosynthetic enzymes arginase, ornithine decarboxylase, and spermidine synthase as potential therapeutic targets in *Leishmania* [33,34,35,36,37,39]. Arginase gene deletion mutants have been generated in *L. mexicana*, *L. major*, *L. amazonensis*, and *L. donovani* [33,35,39,41], and ornithine decarboxylase and spermidine synthase gene deletion mutants have been created in *L. donovani* [34,37]. These mutants demonstrated that the enzymes are each essential in promastigotes for the synthesis of ornithine and polyamines and more importantly, they displayed significantly reduced infectivity compared to wild type parasites. However, although infectivity was reduced, the arginase-deficient mutants in the various *Leishmania* species were still able to establish infections [33,35,36,39], which implies that inhibition of parasite arginase alone may not be a sufficient therapeutic strategy. 

**Figure 3:**
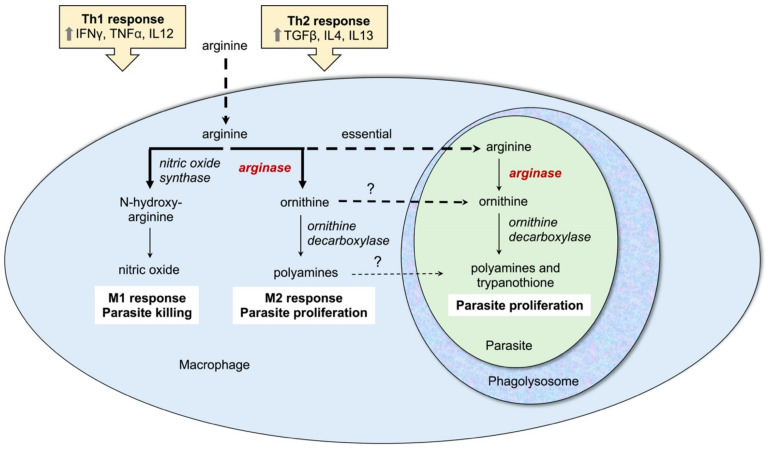
Interplay between host and parasite arginase influence disease outcome. The macrophage, phagolysosome, and intracellular parasite are shown. Indicated in yellow boxes are the opposing Th1 and Th2 responses with associated cytokine production. White boxes denote macrophage polarization towards an M1 or M2 phenotype with resulting parasite killing or proliferation. The conversion of arginine to nitric oxide or polyamines in the macrophage is shown with solid arrows, as is the conversion of arginine to polyamines and trypanothione in the intracellular parasite. Uptake of arginine, ornithine, or polyamines is shown in hashed arrows. The essential uptake of arginine by parasites is indicated; the question marks denote that ornithine and polyamine salvage has not been directly demonstrated in intracellular parasites.

The interplay of host and parasite arginine and polyamine metabolism is crucial for the outcome of *Leishmania* infections and the parasite has the ability to modulate the host arginase activity and immune response. Mammalian cells contain two types of arginase, type I, which is cytosolic, and type II, which is located in the mitochondria. In macrophages arginine is a key amino acid for two competing pathways: arginine can be converted to ornithine by arginase I or alternatively to the potent anti-leishmanial agent nitric oxide by inducible nitric oxide synthase (Figure 3). Polarization of macrophages to the M1 phenotype is associated with nitric oxide production and parasite clearance. This type of response is induced by T-helper type 1 (Th1) cells that produce pro-inflammatory cytokines such as IFNγ, TNFα, and IL12 (Figure 3) [27,31,32,50,51]. However, *Leishmania* parasites have the ability to stimulate differentiation of T cells towards the Th2 subset, which produces the anti-inflammatory cytokines IL4, IL13, and TGFβ. This in turn polarizes macrophages towards the M2 phenotype causing increased arginase activity and parasite proliferation (Figure 3) [27,31,32,50,51]. An increased host arginase expression will reduce the arginine pool and thus lower nitric oxide production. In addition, the increased production of polyamines has been postulated to be used by parasites for their growth [27,31,52], although a comparison of the infectivity phenotypes of *L. donovani* arginase, ornithine decarboxylase and spermidine synthase gene deletion mutants suggests that it may be rather ornithine and not polyamines that are scavenged by intracellular amastigotes [33,34,37]. Murine infectivity models have documented an increased expression of arginase I in susceptible BALB/c mice associated with a TH-2 cell response and higher parasite burden [53,54,55,56,57] and clinical studies in patients have also demonstrated a correlation of disease severity with host arginase expression [32,58,59,60]. Furthermore, inhibition of host arginase reduces parasite loads in infected mice [53,54,56,61]. Together, these studies suggest that both parasite arginase and the regulation of host arginase I play a key role in the balance of life and death of the parasite. 

**Figure 4:**
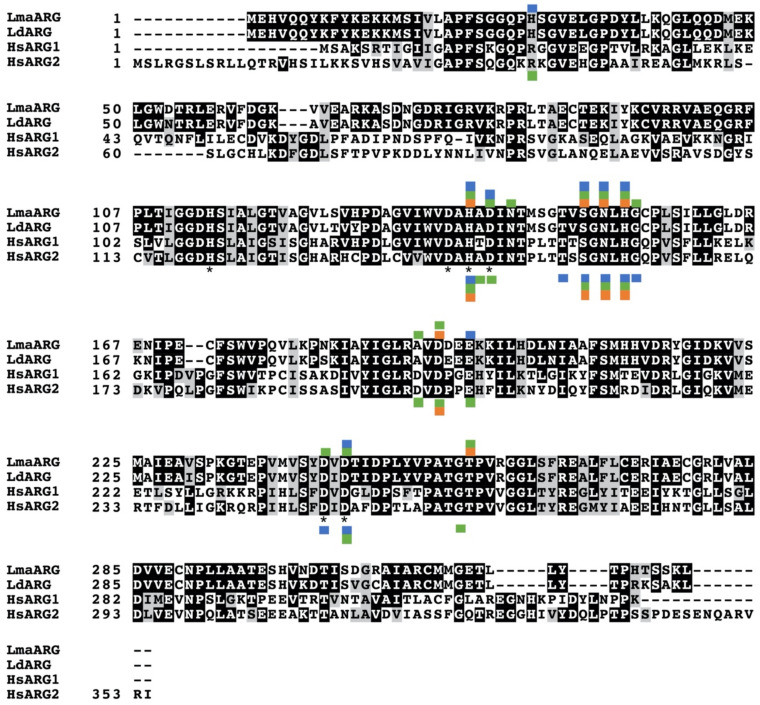
Sequence alignment of leishmanial and human arginases. LmaARG: *L. mexicana amazonensis* arginase, sequence ID AAC95287.1; LdARG: *L. donovani* arginase, sequence ID XP_003864734.1; HsARG1 *Homo sapiens* arginase 1, sequence ID NP_001231367.1; HsARG2: *Homo sapiens* arginase 2, sequence ID NP_001163.1. Sequences were aligned using the Clustal Omega tool. Residues in the multisequence file that were identical or similar between the four sequences were highlighted in black or grey, respectively, using Boxshade. Residues marked with the asterisk were identified as key sites from the first published report of the crystal structure of *L. mexicana* arginase [62]. Boxes above the sequence alignment denote residues where docking studies have indicated that inhibitors bind to leishmanial arginase, whereas boxes below the sequence alignment denote residues where inhibitors bind to host arginase (human or rat). Blue boxes designate sites where flavonols bind (quercetin and fisetin), green boxes show sites where green tea components bind (C, EC, ECG, and GA), and orange boxes indicate sites where cinnamic acids bind (caffeic acid, rosmarinic acid, and chlorogenic acid).

There has been considerable interest recently in inhibitors of the *Leishmania* arginase. Numerous natural products have been identified as having high affinity for the arginase and effectively inhibiting the recombinant enzyme. The availability of the *L. mexicana* arginase crystal structure facilitates computer aided modeling and in silico screening [62,63]. These modeling studies have generally shown that the residues important for inhibitor binding to arginase are strictly conserved between leishmanial and human isoforms (Figure 4). However, although some compounds described here have affinity to both parasite and human arginase, most have selectivity towards the leishmanial arginases. The fact that many of these compounds or their derivatives are active against promastigotes, intracellular amastigotes, and in murine infectivity studies, emphasizes the promise of these compounds for future therapy.

## 4. Natural Products That Target Arginase in *Leishmania*

### 4.1. Quercetin

Quercetin is a polyphenolic flavonoid found in a wide variety of foods including citrus fruits, green leafy vegetables, and green tea [64]. Although most noted for its anti-inflammatory and antioxidant properties, quercetin is also studied for its potential pharmacological applications in anticancer, antiviral, antiplatelet and other treatments [64]. In addition, this compound may exhibit protective effects such as gastroprotective, nephroprotective and neuroprotective outcomes [64]. Quercetin, its derivatives, and related compounds have also been studied for their antiparasitic potential, especially against *Leishmania*.

#### 4.1.1. Inhibition of Recombinant Arginase and Docking Studies

Several studies have demonstrated that quercetin targets the leishmanial arginase (Table 2) [65,66,67]. Quercetin is a mixed inhibitor of recombinant arginase in *L. amazonensis* with an IC_50_ of 3.8 μM while its derivatives quercitrin (IC_50_ 10 μM) and isoquercitrin (IC_50_ 4.3 μM) were shown to be uncompetitive inhibitors [65]. A separate study also confirmed the mixed-type inhibition of *L. amazonensis* arginase by quercetin, as well as by the flavonoid fisetin and luteolin, which demonstrated K_i_ values of 8 μM, 1.9 μM, and 8 μM, respectively [67]. Other quercetin-related compounds such as taxifolin (dihydroquercetin) (IC_50_ 1.6 μM), rutin (quercetin-3-O-rutinoside) (IC_50_ 10.4 μM), and the human metabolite quercetin-3-O-glucuronide (IC_50_ 8.2 μM) were found to target arginase as well [66]. All three studies evaluated inhibition of the recombinant enzyme in vitro and performed in silico docking studies, convincingly demonstrating binding of the flavonoid to leishmanial arginase [65,66,67]. 

#### 4.1.2. Other Cellular Targets of Quercetin

In addition to arginase, other cellular targets of quercetin have been identified in *Leishmania* (Table 2). In silico analysis demonstrated that quercetin has a high affinity for trypanothione synthetase-amidase and trypanothione reductase with docking scores of −4.996 and −4.872, respectively [68]. The potential of quercetin to act as an iron chelator led Sen et al. to investigate its effect on ribonucleotide reductase, an iron dependent enzyme. Their study showed that the flavonoid reduced the activity of ribonucleotide reductase in amastigotes isolated from the spleen of hamsters treated with a combination of quercetin and serum albumin [69]. Quercetin also targets both topoisomerase I and II. The flavonoid was able to induce topoisomerase II-mediated kinetoplast DNA minicircle cleavage in *L. donovani* [70] and in *L. panamensis* [71]. Although quercetin was also active against mammalian topoisomerase II, it was more selective for the *L. panamensis* than the human enzyme with an IC_50_ of 0.6 μM compared to 88.9 μM for the macrophage enzyme [71]. Furthermore, the flavonoid bound to *L. donovani* topoisomerase I and inhibited DNA relaxation, though it appeared less selective, exhibiting an IC_50_ value of 198 μM against the parasite topoisomerase I and 528 μM against the monocyte topoisomerase I [72,73]. In addition, quercetin was found to be an inhibitor of recombinant cathepsin L-like rCPB2.8 [74]. Cathepsin L and B are believed to suppress the antileishmanial immune response and are essential for *Leishmania* virulence [74]. The flavonoid also inhibited *L. infantum chagasi* acetylcholinesterase, an enzyme involved in the formation of the essential membrane component phosphatidylcholine [75]. In silico screening further identified that quercetin has affinity for the guide RNA binding protein, gBP21, which is involved in the editing of mitochondrial gene mRNAs [76]. Together, these findings suggest that quercetin has multiple cellular targets and functions as a promiscuous drug. Such effects can increase its potency and more importantly reduce the possibility for the development of drug resistance.

**Table 2:** Cellular targets of quercetin.

Quercetin Target	*Leishmania* Species	Evidence	Reference
Arginase	*L. amazonensis*	- Inhibition of recombinant enzyme activity - In silico docking studies	[65,66,67]
Trypanothione synthetase-amidase and trypanothione reductase	*L. tropica*	- In silico docking studies	[68]
Ribonucleotide reductase	*L. donovani*	- Reduced enzyme activity in intracellular amastigotes	[69]
Topoisomerase II	*L. donovani* *L. panamensis*	- Inhibition of catalytic activity on minicircle DNA	[70,71]
Topoisomerase I	*L. donovani*	- Binding to recombinant enzyme - Inhibition of plasmid DNA relaxation	[72,73]
Cathepsin L-like rCPB2.8	*L. mexicana*	- Inhibition of recombinant enzyme activity	[74]
gBP21 (RNA editing)	*L. donovani*	- In silico affinity for enzyme	[76]
Acetylcholinesterase	*L. infantum chagasi*	- Inhibition of enzyme activity	[75]

#### 4.1.3. In Vitro Activity against Promastigotes and Axenic Amastigotes

In vitro studies have shown that quercetin is effective against *Leishmania* promastigotes and axenic amastigotes (Table 3). The EC_50_ values reported for the visceralizing species *L. donovani* and *L. infantum* are within similar ranges: 46–86 μM [70,75,77], though one study reported only mild growth inhibitory effects on *L. donovani* promastigotes due to quercetin (the application of 512 μM quercetin reduced parasite growth by 16%) [73]. Similar values have been reported for other species of *Leishmania*, where EC_50_ values of 31.4 μM and 0.7 μM were reported for *L. amazonensis* promastigotes after 24 h [78] and 72 h [79], respectively, and an EC_50_ value of ~48 μM for *L. braziliensis* stationary phase promastigotes after 24 h of incubation [80]. Variations in EC_50_ values between *Leishmania* species are to be expected. Likewise, variations in EC_50_ values within the same species but from separate studies are also not surprising, and differences in the source of the quercetin, as well as in the types of viability assay, likely contribute to this variation. Quercetin is also leishmanicidal against axenic *L. donovani* amastigotes [68,81], as well as against intracellular amastigotes (reviewed in the next section, Table 4 and [70,75,78,79,80]). The exact mechanism by which quercetin induces cell death in these parasites remains to be established, however, quercetin treatment was observed to induce the production of reactive oxygen species and lead to mitochondrial dysfunction in parasites [78,80]. Moreover, *L. donovani* promastigotes treated with quercetin showed apoptotic-like features consistent with programmed cell death [70,80].

#### 4.1.4. In Vitro Efficacy against Intracellular Amastigotes

Quercetin is also effective against intracellular amastigotes in vitro (Table 4). The compound inhibited the growth and replication of *L. amazonensis* amastigotes in peritoneal macrophages derived from Swiss mice with an IC_50_ value of 3.4 μM, an efficacy similar to that of miltefosine, a drug currently used to treat leishmaniasis [82]. A very similar efficacy was observed in a study by Montrieux et al. where the IC_50_ of quercetin against *L. amazonensis* in peritoneal BALB/c macrophages was reported to be 4.3 μM [79]. The EC_50_ for *L. infantum chagasi* amastigotes in murine RAW 264.7 macrophages was 35 μM [75]. Quercetin at concentrations of 48 μM significantly reduced intracellular *L. braziliensis* infectivity in BALB/c peritoneal macrophages [80] and a 70% parasite reduction was observed at 45.5 μM in BALB/c peritoneal macrophages infected with *L. donovani* [70]. Notably, these studies emphasize the high selectivity index of quercetin for *Leishmania* over macrophages, since either no toxicity or toxicity at concentrations significantly higher than those effective against amastigotes were reported [79,80,82].

Despite its high selectivity index, quercetin is a modulator of the host macrophage response, and it is likely that its effect on both the parasite and macrophage contribute to the overall reduction in parasitemia observed in infected macrophages in vitro. The flavonoid has been shown to inhibit mammalian arginase, among other cellular targets [83,84], which likely contributes in part to its immunomodulatory activity (Figure 3). BALB/c peritoneal macrophages infected with *L. braziliensis* showed decreased TNF-α production and increased IL-10 levels when treated with quercetin (48 and 70 μM), although no significant effect on nitric oxide production was observed [80]. Quercetin induced the production of reactive oxygen species in macrophages infected with *L. amazonensis*, while interestingly this was not observed in un-infected macrophages [82], suggesting that quercetin may target a metabolic process in the host that has been modulated by the parasite. Furthermore, quercetin is an iron chelator and induced expression of the transcription factor nuclear factor erythroid 2-related factor as well as heme oxygenase-1 mRNA in *L. braziliensis* infected macrophages, which together decreased the labile iron concentration in macrophages [80]. A different study also demonstrated that quercetin reduces iron acquisition by *L. donovani* in an infected hamster model [69]. Because intracellular *Leishmania* amastigotes depend on iron from the host, the reduced availability of salvageable iron likely contributes to the potency of quercetin against intracellular amastigotes [80].

Taken together, the in vitro infectivity studies demonstrate that quercetin, while exhibiting a low toxicity towards host cells, is active against intracellular *Leishmania* amastigotes and is able to modulate macrophage metabolism towards parasite defense.

#### 4.1.5. Rodent Infectivity Studies

Quercetin proved to be effective in rodent infectivity studies (Table 5). Quercetin at 16 mg/kg body weight was able to control lesion growth and reduced parasite loads in *L. amazonensis* infected mice by 76%, similar to the efficacy observed with Pentostam (62%) [85]. In an attempt to improve the bioavailability of the lipophilic flavonoid, Sousa-Baptista et al. encapsulated quercetin in lipid-core nanocapsules [86] and compared the efficacy of this formulation to free quercetin (un-encapsulated) against *L. amazonensis*-infected BALB/c mice. While daily oral dosing for 51 days with 16 mg/kg of free quercetin reduced parasite lesion sizes and loads by 38 and 71%, respectively, oral daily dosing for 51 days with significantly lower concentrations of quercetin (0.4 mg/kg) in nanocapsules reduced lesion sizes and parasite loads by 64 and 91%, respectively, [86]. In a different study with *L. amazonensis* infected BALB/c mice, quercetin initially caused increased lesion sizes (after 2 and 4 weeks), however, after 5 and 6 weeks of treatment the lesions were smaller than in animals treated with the control drug Glucantime [79]. In the same study, ferulic acid, caffeic acid and rosmarinic acid reduced lesion size in infected mice throughout the study [79]. Quercetin dosed intraperitoneally at 30 mg/kg for 5 days reduced *L. donovani* infections in BALB/c mice by 15.3% [81].

Another strategy that effectively improved the bioavailability of quercetin was the combination of oral delivery of the flavonoid with injections of serum albumin in a hamster infectivity study [69]. Visceral leishmaniasis is associated with a depletion of serum albumin, which may function as transport molecule for quercetin [69]. The delivery of quercetin bound to serum albumin likely increases the availability of the flavonoid compared to its free form [69]. The combination treatment of quercetin and serum albumin increased the reduction of splenic parasite load in golden hamsters from 75% (free quercetin) to 95% (quercetin + serum albumin) in mice infected with *L. donovani* [69]. The level of quercetin in the liver of hamsters was indeed higher when the flavonoid was delivered in combination with serum albumin, demonstrating that the administration improved delivery of the drug to the target tissue [69].

In summary, quercetin was found to be effective in vivo, in both murine and hamster models [69,79,81,85,86]. Formulations that increased the bioavailability of quercetin showed improved efficacy and may be an important aspect in the future development of the flavonoid as a therapeutic treatment.

#### 4.1.6. Pharmacokinetic Considerations for Quercetin

Pharmacokinetics has a key role in the drug discovery and development process and often represents a critical bottleneck in preclinical drug development especially for novel drugs based on natural products. For example, despite the pharmacological efficacy of quercetin towards many disease conditions, its poor pharmacokinetic properties limit its application in the pharmaceutical field [87]. The oral bioavailability of quercetin is very low (approximately 22%), mostly due to its poor water solubility and membrane permeability [88]. Several approaches have been investigated to enhance quercetin’s oral bioavailability to enable its use as a pharmaceutic. The compound’s bioavailability has been improved using creative formulations such as quercetin-loaded lipid liquid crystalline systems [89], quercetin-loaded zein nanoparticles and nanoliposomal encapsulation [90]. In vivo studies with *Leishmania* infected rodents also demonstrated improved efficacy with delivery of quercetin in lipid-core nanocapsules [86] or by combining the oral delivery of the flavonoid with injections of its carrier serum albumin [69].

Quercetin has a very short half-life (less than 30-min) possibly due to rapid metabolism by the liver [88]. Hepatic metabolism of quercetin generates several phase 1 and phase 2 metabolites. The major quercetin metabolites (more than 90%) detected in plasma and urine are glucurono-sulfo conjugates of isorhamnetin (3′-O-methyl quercetin) and of quercetin. Glucuronides of quercetin and its methoxylated forms constitutes the minor metabolites (less than 10%). The metabolites rapidly appear in plasma and reach their highest concentrations in less than 1 h [91]. Several urinary metabolites, of quercetin such as quercetin-3O-glucuronide, have been identified with peak urinary concentration achieved only after 4 h [91].

The disposition of quercetin in humans was examined by Moon et al. in 10 healthy human subjects following daily administration of 1500 mg in three divided doses [92]. The authors reported an average terminal half-life and oral clearance (CL/F) of 3.5 h and 3.5 × 10^4^ L/h, respectively, which were not statistically different between male and female subjects. However, this study suffered from its low sample size and large inter-individual variations. The fraction of quercetin directly excreted in urine is very low (up to 7%) as reported from a phase 1 clinical trial in cancer patients [93].

Quercetin has been reported to competitively inhibit CYP3A4, a drug metabolizing enzyme that is responsible for metabolism of more than 30% of the available drugs on the market [94]. Therefore, quercetin inhibits the elimination, and thus increases plasma concentration, of CYP3A4 substrates such as diltiazem [95]. This should be an important consideration for patients taking quercetin in addition to drugs metabolized by CYP3A4.

Daily human consumption of quercetin ranges from 5–100 mg based on average human consumption of fruits and vegetables [96]. However, individuals that heavily consume quercetin-rich food such as apples and onions may have a daily consumption of up to 500 mg [96]. Most clinical studies that investigated the pharmacological and pharmacokinetic properties of quercetin used daily doses of 50–1000 mg. Daily supplementation of quercetin at doses of 50, 100, and 150 mg to healthy volunteers for 2 weeks achieved peak plasma concentration in the range of 0.4–0.7 μM [97]. Despite the moderate quercetin doses implemented in this study, the achieved plasma concentrations were very close to the reported EC_50_ values from several of the in vitro studies, which suggests the feasibility of orally delivering therapeutic doses of quercetin for the treatment of leishmaniasis.

#### 4.1.7. Summary of Quercetin Studies

In summary, quercetin shows promising anti-leishmanial activity in vitro and in vivo. Several studies have demonstrated that quercetin inhibits recombinant leishmanial arginase and modeling studies validated the affinity of the flavonoid for the enzyme. This activity alone explains its potency as arginase is an essential enzyme. However, the flavonoid is a promiscuous drug as it interferes with additional critical cellular components (Table 2). This promiscuity undoubtedly increases the efficacy of quercetin and in addition should thwart drug resistance. One important advantage of quercetin is that the flavonoid acts on both the parasite and host arginase and modulates the host immune response and metabolism towards parasite defense. While it is toxic to the parasite, it shows no adverse side effects in rodents at concentrations that are effective against *Leishmania* [69,79,81,85,86]. Lastly, although the bioavailability of quercetin is low due to its lipophilic nature, formulations in lipid-core nanocapsules or its co-administration with serum albumin have demonstrated increased in vivo efficacy. Because quercetin is also being developed as a natural product to fight other diseases, development of improved formulations is underway [89,90]. In conclusion, these encouraging results certainly warrant further investigations and hold promise for the development of quercetin or quercetin-related compounds as therapeutic strategies against leishmaniasis.

### 4.2. Fisetin

Fisetin is a flavonoid found in fruits like strawberries and grapes, and in vegetables such as tomatoes and cucumbers [98,99]. It is an antioxidant and anti-inflammatory agent and has anti-diabetic, neuroprotective, and cardioprotective effects in vivo and in vitro [99].

Docking studies have shown higher affinity of fisetin for *L. amazonensis* arginase compared to rat liver arginase I (Figure 4) [67]. Fisetin inhibited the activity of recombinant *L. amazonensis* arginase by 87% at a concentration of 125 μM, but showed no significant inhibition of rat liver arginase I. The flavonoid displayed mixed inhibition of the *Leishmania* arginase with an IC_50_ of 1.4 μM [67].

In vitro studies have shown that fisetin also decreases arginase activity in *L. infantum* promastigotes and is active against both promastigotes and intracellular amastigotes with IC_50_ values of 0.3 and 0.1 μM, respectively [100]. Treatment with fisetin resulted in decreased levels of glutathione within *L. infantum* promastigotes [100]. Since, through the inhibition of arginase, fisetin reduces polyamine and trypanothione levels in the parasite, the reduction of the trypanothione precursor glutathione may further help to reduce trypanothione levels and the parasite’s ability to cope with oxidative stress. Conversely, in infected macrophages, fisetin treatment led to increased glutathione levels, and an increase in catalase and superoxide dismutase activity, but had no effect on arginase activity [100]. Since the toxicity of the antileishmanial drug Glucantime is likely boosted by an increase in macrophage glutathione levels (promoting the reduction of Sb(V) to Sb(III), the latter which is more toxic towards parasites), fisetin has the potential to work synergistically with this drug and improve its overall efficacy [100]. Fisetin may also reduce host toxicity of meglumine antimoniate by reversing oxidative stress [100].

While fisetin was shown to be effective against *L. donovani* axenic amastigotes with an IC_50_ value of 2 μM, surprisingly in vivo infectivity studies in BALB/c mice with 30 mg/kg intraperitoneally for 5 days showed no antileishmanial activity, though quercetin when applied at the same dose showed modest reduction of the parasite liver load (15.3%) [81]. Further studies that explore higher fisetin concentrations, increased and longer dosing regimens, or different routes of administration, are warranted to fully determine the efficacy of this drug.

The pharmacokinetics of fisetin has been established in mice following intraperitoneal administration of 223 mg/kg [101], notably higher concentrations than used in the *Leishmania* study. A peak plasma concentration of 2.5 mg/L was reached after 15 min, followed by a biphasic elimination and a terminal half-life of 3 h [101]. Despite its low plasma concentration in mice, fisetin demonstrates remarkable in vivo biological activity, including an antitumor effect [102]. Such an observation may be explained by the potential accumulation of fisetin and/or its major metabolites (mostly glucuronoid and sulfate conjugates) in the target tissues [103]. A nanoemulsion formulation of fisetin improved its relative bioavailability after intraperitoneal injection by 24-fold as compared to free fisetin [104]. Such a significant enhancement of its bioavailability has enabled the use of lower doses of the fisetin nanoemulsions in Lewis lung carcinoma-bearing mice to achieve an equivalent antitumor activity in comparison to the larger doses of free fisetin. Similarly, nanotechnology-based delivery systems have been developed and demonstrated enhanced bioavailability and improved in vivo activity of fisetin [105].

In summary, in vitro studies were promising for *L. amazonensis, L. infantum*, and *L. donovani*, and despite the lack of efficacy in the mouse model for *L. donovani,* further investigations are warranted.

### 4.3. Polyphenolic Compounds in Green Tea

Green tea, originating in China, has a long history in traditional medicine and is among the most consumed beverages worldwide [106]. It is rich in polyphenols, has antioxidant and anti-inflammatory properties that may be effective to combat cardiovascular disease, hyperlipidemia, obesity, and microbial infections [106]. Catechins present the main polyphenols in tea and include EGCG, ECG, EGC, C and EC [106]. The most abundant and well-studied polyphenol is EGCG. In addition to its function as free-radical scavenger and antioxidant, which is shared among many polyphenols, EGCG is also believed to act as a second messenger and may modulate metabolic enzymes [106].

#### 4.3.1. Inhibition of Recombinant Arginase and Docking Studies

In silico studies established that the four catechins EGCG, ECG, EC, and C have high affinity for the active sites of both arginase (Figure 4) and trypanothione synthetase-amidase [17]. Of the four ligands, EGCG and EC showed the greatest binding affinity to the two enzymes [17]. Docking studies were also performed for the *L. amazonensis* arginase sequence with the catechins EGCG, EC, C, and the polyphenol gallic acid (GA) and demonstrated that EGCG has the highest affinity [107].

In vitro analysis established EGCG, C, EC and GA as potent inhibitors of the recombinant *L. amazonensis* arginase [107]. The polyphenols C and EC are competitive inhibitors with IC_50_ values of 0.8 μM and 1.8 μM, respectively, while EGCG is a mixed inhibitor with an IC_50_ of 3.8 μM, and GA is a noncompetitive inhibitor with an IC50 of 2.2 μM for the leishmanial arginase [107]. The polyphenols were also tested against recombinant rat liver arginase I and exhibited IC_50_ values above 1000 μM, demonstrating that the compounds are potent and selective inhibitors for the leishmanial arginase.

#### 4.3.2. Effect on Morphology and Cellular Metabolism

EGCG induced contractions and cell shrinkage in *L. infantum* promastigotes [17]. Increased hydrogen peroxide production and mitochondrial membrane depolarization was observed in *L. braziliensis* promastigotes [108], and mitochondrial damage was evident in *L. amazonensis* promastigotes when parasites were treated with the compound [109]. These phenotypes are consistent with oxidative stress and may furthermore be indicative of an apoptotic-like cell death. Oxidative stress and apoptosis have also been demonstrated as a consequence of arginine starvation [110], and lend credence to the conjecture that arginase inhibition by EGCG may cause apoptosis. 

#### 4.3.3. In Vitro Activity against Promastigotes

In vitro studies established that several of the polyphenolic compounds in green tea are active against *Leishmania* promastigotes (Table 6). EGCG, EC, C, and ECG have antileishmanial activity against *L. infantum* promastigotes, with EGCG and ECG being the most active compounds with EC_50_ values of 28 μM and 75 μM, respectively [17]. The EC_50_ value for EGCG treatment of *L. donovani*, another visceralizing species, was similar at 42 μM and treatment with GCG achieved an EC_50_ value of 20 μM [81]. Two studies in *L. amazonensis* reported EC_50_ values for EGCG of 63 μM [109] and 36 μM [111]. EGCG proved to be somewhat less effective against *L. tropica*, with an EC_50_ value of 190 μM [112] and the EC_50_ value in *L. braziliensis* was the highest with 278 μM [108]. The polyphenol GA was shown to be effective against *L. amazonensis* with an EC_50_ value of 10 μM [111] and against *L. major* with an EC_50_ of 96 μM [113]. It is intriguing that the EC_50_ values for EGCG are so varied in the different *Leishmania* species with values ranging from 28 to 278 μM. This is likely not due to differences in arginase inhibition, as the enzyme is highly conserved among species, but may relate to the intracellular availability of EGCG amongst the different species of parasite or perhaps in their ability to combat polyamine and trypanothione deficiencies.

#### 4.3.4. In Vitro Efficacy against Intracellular Amastigotes

Several studies have shown that EGCG and other related compounds are effective against intracellular amastigotes (Table 7). EGCG showed impressive potency against intracellular *L. amazonensis* amastigotes in one study with a reported EC_50_ value of 1.6 μM [115]; however, another study reported the EC_50_ value to be 130 μM [116]. The first study used peritoneal macrophages from Swiss mice [115] while the second study evaluated human monocyte THP1 cells [116]. The difference in macrophage cell lines together with variations in the experimental protocols (for example temperature and length of initial incubation with parasites) may account at least in part for the difference in EC_50_ values.

The efficacy against intracellular *L. braziliensis* was reported to be 3.4 μM; while the same study reported an EC_50_ of 278 μM against *L. braziliensis* promastigotes [108]. Al-though this discrepancy between promastigotes and amastigotes is surprising, the higher potency against intracellular amastigotes is more important to promote the compound for further development as a therapeutic agent. One explanation may be that *L. braziliensis* infected peritoneal macrophages, derived from Swiss mice, were able to produce more reactive oxygen species when treated with EGCG compared to infected macrophages that were not treated with the catechin [108]. These pro-oxidative properties of EGCG may contribute to the stimulation of the host defense and add to the higher efficacy of the compound against intracellular amastigotes versus promastigotes.

EGCG was also effective against *L. tropica* amastigotes in J774 macrophages with an EC_50_ of 46 μM [112]. The catechin proved to be more potent than the control drug Glucantime [112]. Interestingly, treatment with both EGCG and Glucantime was more effective in inhibiting intracellular amastigotes than either agent alone [112]. Furthermore, the treatment of infected macrophages with EGCG led to increased expression of IL-12, IL-1bß, metacaspases and nitric oxide synthase with a reduction in IL-10 expression, which is consistent with a Th-1 immune response and defense against parasites [112]. 

The polyphenol GA was found to be effective against *L. major* infected peritoneal macrophages, derived from male and female BALB/c mice, with an EC_50_ of 30 μM [113]. The study also identified immunomodulatory effects of GA on peritoneal macrophages with an increase in lysosomal volume and nitric oxide release [113]. It has been shown previously that GA modulates nitric oxide and cytokine expression in murine RAW 264.7 macrophages infected with *L. major* [117,118]. 

The polyphenol C exhibited an EC_50_ of 287 μM against *L. infantum* infections in RAW 264.7 macrophages and had no effect on nitric oxide production [114]. The infectivity index was below 10 though and raises concerns about host toxicity. 

Importantly, most of these studies found that EGCG was toxic to macrophages only at much higher concentrations than those that were effective against parasites, demonstrating a high selectivity index and promise for further development of green tea polyphenols as therapeutic treatment. 

#### 4.3.5. Murine Infectivity Studies

EGCG and GA show efficacy in vivo. Female BALB/c mice were treated with a topical formulation of 15% EGCG six weeks after infection with *L. amazonensis* [116]. After one week of treatment the edges of the lesions began to flatten, with more significant improvement after 10 additional days [116]. After 18 days, parasite burden was decreased by 80.4% compared to untreated animals, similar to the efficacy of treatment with intraperitoneal Glucantime, which showed 85.1% reduced parasite burden [116]. Another study treated *L. amazonensis* BALB/c mice orally with 30 mg/kg/day and both lesion size and parasite burden were decreased significantly [115]. The potency of oral EGCG in this study was similar to that of intraperitoneal antimony [115]. Yet a third study investigated the effect of ECGC on BALB/c mice infected with *L. braziliensis* [108]. Mice were treated orally with 100 mg/kg/day ECGC, which reduced lesion size and lowered parasite burden by 92% [108]. ECGC showed similar efficacy as Glucantime, which was used as a control drug [108]. GA was tested in a topical formulation in female BALB/c mice infected with *L. major* and demonstrated reduction in lesion size and parasite burden [119]. The study also tested amphotericin B in a topical formulation as control drug and GA showed similar efficacy [119]. Interestingly, the number of parasites remained lower several days after ending the treatment when GA was used; this did not occur with amphotericin B treatment [119]. The authors attribute this to the immune modulatory effect of GA because ex vivo investigation of skin fragments showed macrophage activation in GA treated mice [119]. Importantly, none of these studies observed toxicities in the treated animals, advocating for the safety of EGCG and GA as anti-leishmanial treatments. 

#### 4.3.6. Pharmacokinetic Considerations for Green Tea Polyphenols

Lee et al. studied the pharmacokinetics of green tea polyphenols after drinking the equivalent of two cups of tea [120]. The major catechins detected in the plasma and urine were EGC, EC, EGCG, and ECG as well as their metabolites. Although green tea contains higher amounts of EGCG than other catechins, peak plasma concentration of EGCG was the lowest (approximately 78 ng/mL) despite a similar time to reach peak plasma concentration (approximately 1.5 h). On the other hand, the elimination half-life of EGCG was the longest (3.5 h) compared to other catechins. The pharmacokinetics findings of this study are valuable for designing in vitro experiments for elucidating pharmacological actions and underlying mechanism of green tea catechins. For example, most of the published studies in cell culture and in vitro systems used EGCG at concentrations more than 100-fold its peak plasma concentration [121].

Tea catechins undergo absorption in the small intestine. However, their oral bioavailability has been reported to be poor in both humans and rodents [122]. Due to their hydrophilic nature, the distribution of green tea catechins is expected to be limited, at least initially, to the extracellular space with an expected volume of distribution of 20 L. Elimination of green tea catechin is mostly mediated by phase II metabolizing enzymes in the liver and enterocytes leading to a variety of methylated, sulfated and glucuronidated metabolites [123]. The high concentration of catechin metabolites in both plasma and urine together with the poor bioavailability of the parent catechin led to the belief that the observed biological activity of tea catechin may be attributed to these metabolites.

#### 4.3.7. Summary of Green Tea Polyphenols

In conclusion, the studies suggest that polyphenols found in green tea are potent and selective inhibitors of parasite arginase and have antileishmanial activity against promastigotes and intracellular amastigotes. Murine infectivity studies with EGCG and GA are promising with efficacies that mirror those of currently used anti-leishmanial drugs. The immunomodulatory effects of these compounds likely contribute to their efficacy. Thus far, in vivo infectivity studies have only been performed for cutaneous and cutaneous/mucocutaneous agents and not against *Leishmania* species that cause the visceralizing disease. It is intriguing the EGCG and GA can be formulated both as a topical treatment and for oral therapy. Both would be preferred to the typical intravenous administration of current *Leishmania* drugs. A topical treatment for cutaneous leishmaniasis, even as a supplemental therapy, would be safe and non-invasive. 

### 4.4. Resveratrol

Resveratrol, a polyphenol found in berries, grapes, and peanuts, has been reported to have anti-leishmanial activity against promastigotes and intracellular amastigotes in vitro [124,125,126,127,128]. It is not known if resveratrol inhibits the *Leishmania* arginase, however, it appears to inhibit the mammalian arginase [126,129]. Interestingly, resveratrol may exhibit a synergistic effect with amphotericin B against *Leishmania* promastigotes [126].

### 4.5. Cinnamic Acid Derivatives

Cinnamic acid and its derivatives are polyphenols, that are abundant in plant-based foods such as fruits, vegetables, and whole grains. They have shown to exhibit antioxidant, anticancer, neuroprotective, anti-inflammatory, antidiabetic and antimicrobial properties [130,131].

#### 4.5.1. Inhibition of Recombinant Arginase and Docking Studies

Several natural cinnamic acid derivatives were shown to inhibit the recombinant leishmanial arginase (Table 8). Caffeic, rosmarinic, chlorogenic, and cryptochlorogenic acids, isoverbascoside and verbascoside, inhibited the recombinant *L. amazonensis* arginase with IC_50_ values ranging from 1.5–11 μM [19]. Caffeic, rosmarinic, chlorogenic acids and verbascoside are competitive inhibitors, while cryptochlorogenic acid and isoverbascoside proved to be noncompetitive inhibitors [19]. An independent study also found that verbascoside is a competitive inhibitor of the *L. amazonensis* arginase [132]. Furthermore, the n-butanolic fraction of *Stachytarpheta cayennensis* extract, which contains verbascoside and isoverbascoside at a 7:3 ratio, was shown to be a potent inhibitor of the recombinant *L. amazonensis* arginase [133]. Docking studies performed with the competitive inhibitors, caffeic, rosmarinic, and chlorogenic acids, confirmed the affinity of these compounds to the leishmanial arginase and provide further insight into the molecular basis of arginase inhibition (Figure 4) [19]. Rosmarinic acid and caffeic acid were also investigated for their potential against the recombinant arginase from *L. infantum* and 100 μM rosmarinic acid and caffeic acid inhibited the leishmanial enzyme 71% and 57%, respectively [114].

#### 4.5.2. In Vitro Activity against Promastigotes

Efficacy of caffeic, rosmarinic, and chlorogenic acids against the promastigote stage of several different *Leishmania* species has been demonstrated, however, with varying toxicities even for the same compound within the same species (Table 9). Overall, EC_50_ values varied between 0.6 to above 500 μM. The EC_50_ values for caffeic, rosmarinic, and chlorogenic acids were reported to be 5 μM, 0.7 μM and 0.5 μM for *L. amazonensis* promastigotes, respectively [79]. However, a different study performed with *L. amazonensis* promastigotes found EC_50_ values for caffeic, rosmarinic, and chlorogenic acids to be substantially higher with >500 μM, 61 μM, and >500, respectively [19]. Rosmarinic acid and caffeic acid were found to be effective against *L. infantum* promastigotes with EC_50_ values of 57 and 61 μM, respectively [114]. A study investigating the efficacy of these compounds in *L. donovani* promastigotes found EC_50_ values for caffeic acid, rosmarinic acid, and chlorogenic acid of 59 μM, 16 μM, and 54 μM, respectively [20].

Isoverbascoside and verbascoside were found to be effective against *L. amazonensis* promastigotes with EC_50_ values of 14 μM and 19 μM, respectively [19,132]. The n-butanolic fraction of *S. cayennensis* extract, which contains verbascoside and isoverbascoside, exhibited an EC_50_ of 51 μg/mL and importantly the toxicity toward *L. amazonensis* promastigotes could be rescued with ornithine supplementation, demonstrating that the mechanism of action of verbascoside and isoverbascoside is indeed via arginase inhibition [133].

Another study found that synthetic cinnamic acid derivatives in liposomal formulations were more efficient than derivatives not delivered in liposomes, again demonstrating that these special formulations may increase the delivery of lipophilic compounds in *L. amazonensis* promastigotes [134].

**Table 9:** Inhibition of *Leishmania* promastigotes with cinnamic acid derivatives.

Compound	Parasite Species	EC_50_	Reference
Caffeic acid	*L. amazonensis*	5.2 μM after 72 h	[79]
	*L. amazonensis*	>500 μM after 72 h	[19]
	*L. infantum*	61 μM	[114]
	*L. donovani*	59 μM	[20]
Rosmarinic acid	*L. amazonensis*	0.6 μM after 72 h	[79]
	*L. amazonensis*	61 μM after 72 h	[19]
	*L. infantum*	57 μM	[114]
	*L. donovani*	16 μM	[20]
Chlorogenic acid	*L. amazonensis*	0.5 μM after 72 h	[79]
	*L. amazonensis*	>500 μM after 72 h	[19]
	*L. donovani*	54 μM	[20]
Isoverbascoside	*L. amazonensis*	14 μM after 24 h	[19]
Verbascoside	*L. amazonensis*	19 μM after 72 h	[19]
	*L. amazonensis*	19 μM	[132]
n-butanolic fraction of *S. cayennensis* extract	*L. amazonensis*	51 μg/mL after 72 h	[133]

EC_50_ (half maximal effective concentration).

#### 4.5.3. In Vitro Efficacy against Intracellular Amastigotes

Caffeic acid and rosmarinic acid proved to be effective against *L. infantum* in RAW 264.7 macrophages with EC_50_ values of 22 μM and 8 μM (Table 10) [114]. The compounds also showed higher toxicity for *Leishmania* parasites than macrophages with selectivity indices of 56 and 62 for caffeic acid and rosmarinic acid, respectively [114]. Furthermore, caffeic acid but not rosmarinic acid increased nitric oxide production in infected macrophages [114]. Caffeic acid has previously been shown to affect cytokine expression and decrease arginase I activity in tumor associated macrophages [135]. The modulation of the host response likely contributes to the anti-leishmanial activity of caffeic acid. 

Caffeic, rosmarinic, and chlorogenic acids demonstrated even higher efficacy against *L. amazonensis* amastigotes in macrophages derived from female BALB/c mice with IC_50_ values of 16 μM, 4.8 μM, and 5.3 μM, respectively (Table 10) [79]. Caffeic acid and rosmarinic acid also demonstrated promising selectivity indices of 11 and 20, respectively, while the selectivity index for chlorogenic acid was below10, showing some toxicity towards the host at the effective dose [79].

Verbascoside exhibited activity against intracellular *L. amazonensis* amastigotes in RAW 264.7 macrophages with an EC_50_ value of 32 μM; however, the selectivity index was below 10 indicating toxicity towards host cells [136]. The compound inhibited arginase activity in promastigotes efficiently but only marginally in macrophages, with 100% and 17% inhibition, respectively [136]. Importantly, the supplementation of 5 mM ornithine, the product of the arginase reaction, to the media of infected macrophages reverted the toxicity of verbascoside [136]. Furthermore, verbascoside did not modulate cytokine expression or nitrate production [136], Thus, this compound is one of the few natural products that has been demonstrated to act specifically through the inhibition of parasite arginase. The n-butanolic fraction of *S. cayennensis* extracts (containing verbascoside and isoverbascoside) was also active against intracellular amastigotes in RAW 264.7 macrophages with an EC_50_ value of 32 μg/mL and again supplementation with ornithine mitigated toxicity underscoring the specificity of these compounds for parasite arginase [133].

A series of cinnamic acid derivates with isobenzofuranone and 1,2,3-triazole functionalities were tested against *L. braziliensis* promastigotes and intracellular amastigotes and found to be more effective against amastigotes [137]. Two compounds were effective with over 80% toxicity at 10 μM concentrations against amastigotes in RAW264.7 macrophages and demonstrated similar efficacy as the control drug amphotericin B [137]. The compounds were not toxic in macrophages at 10 μM concentrations demonstrating selectivity towards intracellular parasites [137]. Transmission electron microscopy studies using one of these compounds suggested that damage in cytokinesis and possibly apoptosis occurred in intracellular amastigotes [137]. A series of cinnamic acid conjugates of chloroquine proved effective against *L. infantum* promastigotes and intracellular amastigotes (bone marrow derived macrophages from BALB/c mice) with IC_50_ values ranging from 3–22 μM for promastigotes and 1–10 μM for amastigotes [138]. However, with the exception of one compound (with a selectivity index of 21), most of the selectivity indices were below 10 demonstrating that the compounds exhibited host toxicity [138].

#### 4.5.4. Murine Infectivity Studies

Caffeic acid and rosmarinic acid demonstrated efficacy against *L. amazonensis* in infected female BALB/c mice and were able to reduce lesion size and parasite burden when injected into the lesions five times at 30 mg/kg every 4 days [79]. Importantly, the compounds proved to be more effective against the infection than the reference drug Glucantime [79].

A cinnamic acid bornyl ester (bornyl 3-phenylpropanoate) was found to be effective against *L. major* infections in female BALB/c mice and at 50 mg/kg/day prevented swelling and reduced parasitemia [139]. In promastigotes the compound resulted in mitochondrial swelling and loss of mitochondrial potential [139].

#### 4.5.5. Pharmacokinetic Considerations for Cinnamic Acid Derivatives

The pharmacokinetics, especially oral bioavailability, of cinnamic acid derivatives demonstrate significant interindividual variability not only due to variation in daily consumption from different natural sources, but also due to diverse molecular structure, metabolism and absorption from the gut [140]. Several published studies using in situ or ex vivo absorption models reported that cinnamic acid derivatives in their ester form are slowly absorbed throughout the gastrointestinal tract compared to their free forms [141]. Ferulic and sinapic acids were the most abundant cinnamic acid derivatives detected in human plasma with peak plasma concentration achieved at 1- and 3-h following administration of high-bran cereal, respectively [142]. The most commonly reported mechanisms for transcellular absorption are passive transcellular diffusion, facilitated transport, and active transport [143]. Calculation of area under the curve (AUC) following oral administration of cinnamic acid derivatives by oral gavage revealed relative bioavailability of cinnamic acid derivatives in the following order: chlorogenic acid < rosmarinic acid < caffeic acid < ferulic acid < *p*-coumaric acid [144].

The uptake and distribution of cinnamic acid derivatives is highly dependent on their physicochemical properties and metabolism. The distribution kinetics of most cinnamic acid derivatives demonstrated low concentration in the liver, possibly due to limited first-pass metabolism, and greater accumulation in the kidneys [145]. The distribution pattern of cinnamic acid derivatives plays a major role in their health benefits and therapeutic uses. For example, efficient distribution of caffeic acid in the skin of hairless mice model likely contributes to its suppressive effect on the generation of UVA-induced reactive oxygen species following oral administration [146].

The major metabolites of cinnamic acid derivatives detected in rat plasma are phase 2 conjugates mostly through conjugation with glucuronide, sulfate and sulfoglucuronide in the liver [147]. A plethora of evidence indicates that the phase 2 conjugates strengthen the biological activity of cinnamic acid derivatives. A minor metabolic pathway includes oxidation to benzoic acid followed by further conversion to hippuric acid derivatives [148]. Most of the cinnamic acid derivatives conjugate metabolites undergo excretion in the urine and bile [149].

#### 4.5.6. Summary of Cinnamic Acid Derivatives

Cinnamic acid derivatives show promise against promastigotes, intracellular amastigotes and in murine infectivity studies. Several compounds were tested against promastigotes of *L. amazonensis, L. infantum,* and *L. donovani* with EC_50_ values typically in the range of 0.6–61 μM (Table 9) and against intracellular amastigotes of *L. infantum* and *L. amazonensis* with EC_50_ values between 4.8 and 32 μM (Table 10). While caffeic acid modulates the host immune response, this does not appear to be case for rosmarinic acid [114]. Verbascoside is the only natural product that has been shown unequivocally to target the parasite arginase specifically: it does not reduce arginase activity in macrophages and ornithine supplementation reverts the growth inhibition of intracellular *L. amazonensis* amastigotes [133,136]. Most promising is that caffeic acid and rosmarinic acid were effective against *L. amazonensis* in murine infectivity studies and furthermore demonstrated higher efficacy than the reference drug Glucantime [79]. Together, these studied demonstrate promise for the cinnamic acid derivatives and more in vivo infectivity studies should be encouraged for further development toward therapeutic treatment strategies.

### 4.6. Senna Spectabilis Extracts and Compounds

*Senna spectabilis* is a species of tree originating from tropical regions of the Americas, whose common name is whitebark senna. The piperidine alkaloids cassine, spectaline, 3-O-acetylcassine, and 3-O-acetylspectaline that can be isolated from *S. spectabilis* have the potential to be useful against *Leishmania* infections due to their ability to inhibit the leishmanial arginase [150,151]. In silico studies demonstrated that spectaline and 3-O-acetylspectaline bound to *L. amazonensis* arginase tighter than cassine and 3-O-acetylcassine, likely due to extra interactions with side chains [151]. Similarly, spectaline and 3-O-acetylspectaline were more effective against *L. amazonensis* promastigotes in in vitro studies and achieved IC_50_ values of 49 μM and 71 μM, respectively [151]. These were smaller than the EC_50_ concentrations determined against Vero cells, which were 205 μM and 744 μM for spectaline and 3-O-acetylspectaline, respectively, which demonstrate low host toxicity. Cassine and spectaline, as well as a crude ethanolic extracts and fractions, have also been evaluated for activity against *L. major* promastigotes, [150]. The two alkaloids combined were found to have an IC_50_ of 24.9 μg/mL [150]. The crude extract and most of the fractions were not as effective, having IC_50_ values over 100 μg/mL. There were two fractions, however, which partitioned with n-butanol and dichloromethane, referred to as FL-Bu and FL-DCM respectively, which had smaller IC_50_ values than the purified alkaloids. The values were 1.6 μg/mL for FL-Bu and 0.6 μg/mL for FL-DCM, which suggest they may be more potent than the alkaloids alone. All of the compounds were also tested in J774 murine macrophages, and were not found to be cytotoxic [150]. These studies are promising, however, studies against intracellular parasites and infectivity studies are necessary to evaluate therapeutic promise.

### 4.7. Extracts from Sambucus Ebulus and Urtica Dioica

*Sambucus ebulus*, commonly known as dwarf elder or elder, has long been used in traditional herbal medicine to treat infections, wounds, joint pains and may have potential for the treatment of metabolic disorders and cancer [152]. The herb contains several phytochemicals like flavonoids, phenols, and lectins, that have anti-oxidant, anti-inflammatory, and anti-microbial effects [152]. A comparison of *S. ebulus* extracts from leaf and fruit extracts found that both inhibited *L. major* promastigotes and intracellular amastigotes in vitro, with the leaf extracts being more effective (EC_50_ values of 157 μg/mL and 265 μg/mL, respectively) and inducing apoptosis in the parasite [153]. Efficacy was also tested in BALB/c mice infected with *L. major* parasites by intramuscular or intralesional injections of 100–200 mg/kg of different extracts [154]. Intralesional administration of the extract proved to be more efficacious than intramuscular injections [154]. Lesion size, parasite burden, and arginase activity were reduced with a concomitant increase in nitric oxide and IFN- γ [154]. Thus, although the mechanism of action in the parasite has not been elucidated, the host arginase appears to be targeted by the *S. ebulus* extracts. These studies are particularly promising as they show efficacy in the animal model with no host toxicity. A closer investigation into which compounds are effective and into the molecular mechanism of parasite inhibition is merited.

*Urtica dioica*, stinging nettle, has been reported to have a wide range of effects, including anti-inflammatory and immune stimulatory properties [155]. Extracts from *U. dioica* were found to have in vitro anti-leishmanial activity against *L. major* promastigotes and intracellular amastigotes and furthermore reduced footpad lesions and parasite loads in BALB/c mice with no toxicity to the host [156]. Arginase activity was decreased, along with a decrease in IL-4 levels, while IFN-γ and nitric oxide production was increased. The mechanism of action by which the extract kills *Leishmania* parasites has not been elucidated.

The extracts from *S. ebulus* and *U. dioica* are promising for the development of therapeutic treatment due to their efficacy in the animal model. Although they are effective against *Leishmania* parasites, the mechanism of action in parasites has not been studied. More is known about their effect on the infected host. Both *S. ebulus* and *U. dioica* extracts modulate the host response by reducing arginase activity with a concomitant increase in nitric oxide and IFN-γ, suggesting a shift towards a Th1/M1 response. Importantly, no host toxicity has been observed in the murine infectivity studies. The combination of host modulation and anti-parasitic activity make these extracts particularly effective and should also slow the emergence of drug resistance.

## 5. Future Outlook for Arginase Inhibitors as Anti-Leishmanial Therapeutics

**Figure 5:**
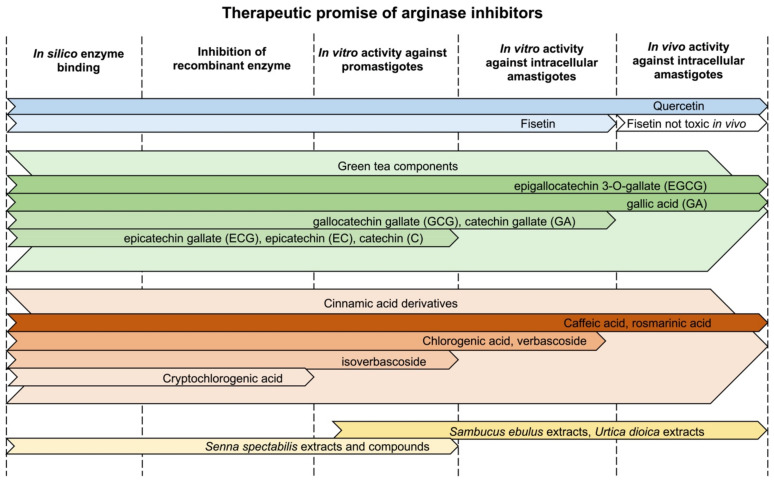
Therapeutic promise of arginase inhibitors. Colored arrows indicate if compounds have been tested in silico, were evaluated against the recombinant *Leishmania* arginase, exhibited toxicity towards promastigotes, demonstrated in vitro activity against intracellular amastigotes, or displayed in vivo efficacy.

The natural products summarized in this review are at various stages of therapeutic validation, with several showing efficacy in rodent infectivity models (Figure 5). The most promising compound is quercetin with five independent rodent infectivity studies that demonstrated its therapeutic ability to reduce parasite loads [69,79,81,85,86]. Murine infectivity studies were performed with *L. donovani* and *L. amazonensis* and a hamster infectivity study was implemented with *L. donovani* (Table 5) [69,79,81,85,86]. The efficacy of quercetin was similar to that of the control drug Pentostam [85]. Two of the studies used special formulations to increase the delivery of the hydrophobic compound and both approaches increased the efficacy of quercetin [69,86]. This is likely an important strategy for most natural products because they tend to be lipophilic. The green tea polyphenols also show encouraging results. EGCG and GA have been tested in mice infected with *L. amazonensis*, *L. braziliensis* or *L. major* and resulted in significant reductions in lesion size and parasite burden [108,115,116,119]. EGCG was evaluated in three studies; one study applied a topical formulation [116] and two studies used oral delivery [108,115]. Importantly, EGCG proved to be more effective than intraperitoneal antimony [108,115]. Among the cinnamic acid derivatives, caffeic acid and rosmarinic acid proved to be more effective in murine infectivity studies than the control drug Glucantime [79]. Other compounds have been tested in vitro and further studies in animals are necessary to evaluate their therapeutic potential. 

Interestingly, docking studies across multiple labs have shown that many of the natural products outlined in this review bind to highly conserved residues within arginase. In fact, many appear to utilize the same residues (e.g., H139, S150, N152, ad H154) independent of their structural classification (Figure 4). Even inhibitors within the same structural class appear to bind in different orientations within leishmanial arginase. For example, the catechol of caffeic acid has been shown to interact with S150; however, this same residue was shown to interact with the α-β unsaturated carbonyl oxygen in rosmarinic acid [19]. Interestingly, despite a strong conservation of key residues between the leishmanial and human arginases, not all of the natural products inhibit both enzymes. For example, quercetin and caffeic acid are potent inhibitors of recombinant host arginase I [83,84,157]. The polyphenols EGCG, C, EC and GA, however, exhibited IC_50_ values above 1000 μM and thus had only moderate inhibitory effects on the mammalian arginase I [107]. Assays with the recombinant *Leishmania* arginase from several labs have further confirmed the ability of these natural products to inhibit the parasite enzyme [19,65,67,107,114,132]. While quercetin, fisetin, and ECGC are mixed inhibitors, C, EC, caffeic acid, rosmarinic acid, chlorogenic acid, and verbascoside are competitive inhibitors [19,65,67,107,114,132]. Three of the compounds reviewed, GA, cryptochlorogenic, and isoverbascoside, have been characterized as noncompetitive inhibitors [19].

Inhibition of parasite arginase results in the reduced production of polyamines, trypanothione and hypusinated eIF5A, all of which are essential metabolites, and their depletion would cause parasite death. However, inhibition of parasite arginase alone is likely not a sufficient strategy because arginase gene deletion mutants are still able to establish an infection, albeit significantly less so than wild type parasites [33,35,36,39]. This suggests that many of the natural compounds have additional cellular targets. Indeed, quercetin, has been shown to have multiple targets in the parasite (Table 2), likely contributing to its efficacy and decreasing the risk of drug resistance. Moreover, many of the tested compounds also have immuno-stimulatory effects. For example, quercetin inhibits mammalian arginase, has immunomodulatory effects, and may sequester iron away from the parasite [69,80,82,83,84] and some of the green tea compounds and cinnamic acid derivatives are inducers of nitric oxide production [113,114,117,118]. Dual targeting of the host and parasite metabolism may increase the potency of these agents and reduce the opportunity for drug resistance. Though any therapy targeting the host has the risk of negative side effects, notably, the studies summarized here show little toxicity to the host cells and many of these compounds are in use or are being developed for treating various human diseases. When considering the length of time it takes to bring a drug treatment to the market, natural products offer an attractive alternative to pharmaceutical drugs for the treatment of leishmaniasis since natural products, such as those that can be classified as dietary supplements, tend to face fewer regulatory roadblocks. In the United States for example, Dietary Supplement Health and Education Act (DHSEA), places the responsibility for ensuring pre-market product safety, truthful product label information, and FDA registration on the manufacturer and not on a regulatory body. Interestingly, dietary supplement manufacturers are not responsible for providing the FDA with the evidence substantiating the safety or efficacy of their products before or after they market their products. This is obviously quite different from the pharmaceutical drug approval pipeline and allows dietary supplements in the United States to hit the market more quickly. However, this lack of regulation can be associated with potential risks as the process is certainly less thorough. A shortened approval process coupled with evidence that the natural products presented in this review possess a safe toxicity profile suggests further investigations with these compounds as anti-leishmanials is warranted. It might be possible to piggyback on clinical studies performed for other disease states, because safety profiles and pharmaceutical formulations are either already available or being developed.

The studies described here have shown efficacy within an impressive array of *Leishmania* species, including *L. major* and *L. tropica*, which cause cutaneous leishmaniasis, *L. amazonensis* and *L. braziliensis* that can precipitate both cutaneous or mucocutaneous leishmaniasis, and *L. donovani* and *L. infantum*, agents of visceral leishmaniasis. Treatment modalities of topical or systemic delivery will have an impact for therapeutic development of these agents. Particularly for cutaneous leishmaniasis, the development of an ointment or cream, even to only reduce ulceration or scarring, could be of benefit. Furthermore, the limited number of current therapeutic options necessitates monotherapy. The combination of two or more drugs, or the addition of supportive therapy would be a tremendous benefit to the treatment of leishmaniasis. Here, natural products could have an impact on reducing symptoms by working synergistically with existing medications.

## 6. Concluding Remarks

The natural products reviewed here target the leishmanial arginase, are toxic to promastigotes and intracellular amastigotes, and some have demonstrated efficacy in rodent infectivity studies. Many of the compounds have additional targets in *Leishmania* and also stimulate the host’s defense mechanisms. This dual modality has the potential to increase efficacy and thwart the development of drug resistance. The fact that many of the natural compounds are also being developed for the treatment of other diseases is of advantage as safety profiles and more efficacious formulations are being developed and strategies targeting leishmaniasis could take advantage of these efforts. Overall, natural products targeting the leishmanial arginase offer tremendous promise and further work into pre-clinical and clinical trials is certainly warranted.

## Figures and Tables

**Table 1:** Relevant *Leishmania* species.

*Leishmania* Species	Type of Leishmaniasis	Geographical Distribution	Old vs. New World
*L. major*	Cutaneous	West Africa to Central Asia	Old world
*L. tropica*		Eastern Mediterranean, Middle East, North India, Afghanistan, and northeast and South Africa	
*L. donovani*	Visceral	South Asia, East Africa	
*L. infantum/* *L. infantum* *chagasi*		Middle East, Afghanistan, Iran, Pakistan	
*L. braziliensis*	Cutaneous, Mucosal	South America	New world
*L. amazonensis*		South America	
*L. mexicana*	Cutaneous	South America	
*L. infantum/* *L. infantum chagasi*	Visceral	Mexico and Central and Latin America	

**Table 3:** In vitro anti-leishmanial activity of quercetin.

*Leishmania* Species	EC_50_ Promastigotes or Efficacy	EC_50_ Axenic Amastigotes	Reference
*L. amazonensis*	31 μM (48 h)		[78]
	0.7 μM (72 h)		[79]
*L. donovani*		3.3 μM	[81]
	46 μM (24 h)		[70]
	64 μM		[77]
	16% growth inhibition in 512 μM quercetin		[73]
*L. infantum chagasi*	86 μM (24 h)		[75]
*L. tropica*	603 μM (72 h)	454 μM (72 h)	[68]
*L. braziliensis*	≤50% viability in 48 μM quercetin at 24 h		[80]

EC_50_ (half maximal effective concentration) (h) hours of incubation with compound before EC_50_ was assessed.

**Table 4:** In vitro activity of quercetin against intracellular amastigotes.

*Leishmania* Species	Macrophages Derived from	EC_50_ or Efficacy	Reference
*L. amazonensis*	Peritoneal Swiss mice	3.4 μM	[82]
	Peritoneal female BALB/c mice	4.3 μM	[79]
*L. braziliensis*	Peritoneal female BALB/c mice	Statistically significant reduction of infectivity at 48 μM and 70 μM concentrations	[80]
*L. donovani*	Peritoneal BALB/c mice	70% reduction of intracellular amastigotes at 46 μM	[70]
*L. infantum chagasi*	RAW 264.7	35 μM	[75]

EC_50_ (half maximal effective concentration).

**Table 5:** In vivo efficacy of quercetin.

*Leishmania* Species	Rodent	Route of Administration and Efficacy	Reference
*L. donovani*	Golden hamsters	- Quercetin delivered (orally) free or in combination with serum albumin (injected); quercetin doses: 5 to 50 mg/kg - Free quercetin: 75% reduction in splenic parasite load - Quercetin + serum albumin: 95% reduction in splenic parasite load	[69]
	BALB/c mice	- Quercetin dose (intraperitoneally): 30 mg/kg (5 doses) - 15% inhibition of liver parasite load	[81]
*L. amazonensis*	BALB/c mice	- Quercetin and lipid-core nanocapsules containing quercetin (orally); 16 mg/kg (51 doses) - Free quercetin: Lesion size reduced by 38%, parasite loads reduced by 71% - Quercetin nanocapsules: Lesion size reduced by 64%, parasite loads reduced by 91%	[86]
	BALB/c mice	- Quercetin dose (intragastric gavage): 16 mg/kg (30 doses) - reduced parasite burden by 76%	[85]
	BALB/c mice	- Quercetin dose (intralesion injections): 30 mg/kg (5 doses) - reduced lesion size after 5 and 6 weeks	[79]

**Table 6:** In vitro activity of green tea polyphenols against promastigotes.

*Leishmania* Secies	EC_50_						Reference
	EGCG	ECG	GCG	C	EC	GA	
*L. infantum*	28 μM	75 μM		94 μM	212 μM		[17]
				395 μM			[114]
*L. donovani*	42 μM		20 μM				[81]
*L. amazonensis*	63 μM						[109]
	37 μM			145 μM		10 μM	[111]
*L. braziliensis*	278 μM						[108]
*L. tropica*	190 μM						[112]
*L. major*						97 μM	[113]

EC_50_ (half maximal effective concentration), EGCG (Epigallocatechin-3-gallate), ECG (Epicatechin gallate), GCG (Gallocatechin gallate), C (Catechin), EC (Epicatechin), GA (Gallic acid).

**Table 7:** In vitro activity of green tea polyphenols against intracellular amastigotes.

*Leishmania* Species	EC_50_					Macrophages Derived from	Reference
	EGCG	GCG	GC	C	GA		
*L. amazonensis*	130 μM	148 μM	213 μM			THP1	[116]
	1.6 μM					Peritoneal Swiss mice	[115]
*L. braziliensis*	3.4 μM					Peritoneal Swiss mice	[108]
*L. tropica*	46 μM					J774	[112]
*L. major*					30 μM	Peritoneal BALB/c mice	[113]
*L. infantum*				287 μM		RAW 264.7	[114]

EC_50_ (half maximal effective concentration), EGCG (Epigallocatechin-3-gallate), GCG (Gallocatechin gallate), GC (Catechin gallate), C (Catechin), GA (Gallic acid).

**Table 8:** Inhibition of recombinant *L. amazonensis* arginase by cinnamic acid derivatives.

Compound	Parasite Species	IC_50_ Ki % Inhibition	Type of Inhibition	Reference
Caffeic acid	*L. amazonensis*	IC_50_ 1.5 μM Ki 0.5 μM	Competitive	[19]
	*L. infantum*	57% inhibition at 100 μM concentration		[114]
Rosmarinic acid	*L. amazonensis*	IC_50_ 2.1 μM Ki 1.8 μM	Competitive	[19]
	*L. infantum*	71% inhibition at 100 μM concentration		[114]
Chlorogenic acid	*L. amazonensis*	IC_50_ 8.3 μM Ki 5 μM	Competitive	[19]
Cryptochlorogenic acid	*L. amazonensis*	IC_50_ 11 μM Ki 12.3 μM	Noncompetitive	[19]
Isoverbascoside	*L. amazonensis*	IC_50_ 2.3 μM Ki 1 μM	Noncompetitive	[19]
Verbascoside	*L. amazonensis*	Ki 0.7 μM	Competitive	[19]
	*L. amazonensis*	Ki 0.7 μM	Competitive	[132]
N-butanolic fraction of *S. cayennensis* extract	*L. amazonensis*	IC_50_ 1.2 μg/mL		[133]

IC_50_ (half maximal inhibitory concentration), Ki (inhibition constant).

**Table 10:** Inhibition of intracellular amastigotes by cinnamic acid derivatives.

Compound	Parasites Species	EC_50_	Macrophage	Reference
Caffeic acid	*L. infantum*	22 μM	RAW 264.7	[114]
	*L. amazonensis*	16 μM	Peritoneal, from female BALB/c mice	[79]
Rosmarinic acid	*L. infantum*	7.9 μM	RAW 264.7	[114]
	*L. amazonensis*	4.8 μM	Peritoneal, from female BALB/c mice	[79]
Chlorogenic acid	*L. amazonensis*	5.3 μM	Peritoneal, from female BALB/c mice	[79]
Verbascoside	*L. amazonensis*	32 μM	RAW 264.7	[136]
n-butanolic fraction of *S. cayennensis* extract	*L. amazonensis*	32 μg/mL	RAW 264.7	[133]

EC_50_ (half maximal effective concentration).

## Data Availability

Not applicable.

## Abbreviations

C	Catechin
EC	Epicatechin
ECG	Epicatechin gallate
EGC	Epigallocatechin
EGCG	Epigallocatechin-3-gallate
eIF5A	Eukaryotic translation initiation factor 5A
FL-Bu	n-butanol fraction
FL-DCM	Dichloromethane fraction
GA	Gallic acid
GCG	Gallocatechin gallate
IFN	Interferon
IL	Interleukin
Th1	T-helper type 1
TNF	Tumor necrosis factor
